# Arginase-II Deficiency Extends Lifespan in Mice

**DOI:** 10.3389/fphys.2017.00682

**Published:** 2017-09-08

**Authors:** Yuyan Xiong, Gautham Yepuri, Jean-Pierre Montani, Xiu-Fen Ming, Zhihong Yang

**Affiliations:** ^1^Division of Physiology, Cardiovascular and Aging Research, Department of Medicine, University of Fribourg Fribourg, Switzerland; ^2^National Center of Competence in Research “Kidney.CH” Fribourg, Switzerland

**Keywords:** aging, arginase, lifespan, mTOR, S6K1, p66^Shc^, p16^INK4a^

## Abstract

The mitochondrial arginase type II (Arg-II) has been shown to interact with ribosomal protein S6 kinase 1 (S6K1) and mitochondrial p66^Shc^ and to promote cell senescence, apoptosis and inflammation under pathological conditions. However, the impact of Arg-II on organismal lifespan is not known. In this study, we demonstrate a significant lifespan extension in mice with Arg-II gene deficiency (Arg-II^−/−^) as compared to wild type (WT) control animals. This effect is more pronounced in the females than in the males. The gender difference is associated with higher Arg-II expression levels in the females than in the males in skin and heart at both young and old age. Ablation of Arg-II gene significantly reduces the aging marker p16^INK4a^ levels in these tissues of old female mice, whereas in the male mice this effect of Arg-II deficiency is weaker. In line with this observation, age-associated increases in S6K1 signaling and p66^Shc^ levels in heart are significantly attenuated in the female Arg-II^−/−^ mice. In the male mice, only p66^Shc^ but not S6K1 signaling is reduced. In summary, our study demonstrates that Arg-II may play an important role in the acceleration of aging in mice. Genetic disruption of Arg-II in mouse extends lifespan predominantly in females, which relates to inhibition of S6K1, p66^Shc^, and p16^INK4a^. Thus, Arg-II may represent a promising target to decelerate aging process and extend lifespan as well as to treat age-related diseases.

## Introduction

Aging is a process of progressive decline or loss in physiological integrity of a cell or organism with increasing chronological time, leading to impaired function and death (Lopez-Otin et al., 2013). Aging is the major risk factor for most human diseases, such as cardiovascular diseases, metabolic diseases, cancer, and neurodegenerative disorders (Lamming et al., 2012). Although aging is generally considered as an inevitable process, recent studies provide convincing evidence that the aging process is modifiable. Genetic or pharmacological interventions of specific genes or signaling pathways are able to modulate aging processes and regulate lifespan in multiple organisms (Ocampo et al., 2016). Numerous molecules or pathways, including mechanistic target of rapamycin (mTOR) and the downstream kinase ribosomal protein S6 kinase 1 (S6K1), the mitochondrial p66^Shc^, and p16^INK4a^, are not only the biomarkers of cell or organism aging, they are also identified as important regulators of aging and lifespan in several model organisms (Migliaccio et al., 1999; Selman et al., 2008; Kanfi et al., 2012; Baker et al., 2016). Indeed, studies demonstrate that a sustained hyperactive mTOR/S6K1 pathway promotes cellular senescence and organism aging and participates in the pathogenesis of cardiovascular and metabolic diseases (Rajapakse et al., 2011; Yang and Ming, 2012; Saxton and Sabatini, 2017). Accordingly, inhibition of mTOR/S6K1 pathway in model organisms including invertebrate animals and rodent mice extends lifespan (Selman et al., 2008; Harrison et al., 2009; Bitto et al., 2016; Pan and Finkel, 2017). In addition, the mitochondrial redox enzyme p66^Shc^ that plays an important role in mitochondrial reactive oxygen species (ROS) generation, is implicated in the acceleration of aging and age-associated diseases (Camici et al., 2015; Giorgio et al., 2016). Interestingly, cellular senescence accumulates in aged organisms and elimination of p16^INK4a^ expressing senescent cells in mice mitigates tissue degeneration and has been shown to extend healthy lifespan (Baker et al., 2016). All these studies demonstrate features of aging plasticity. Targeting these mechanisms involved in accelerating the aging process could extend lifespan and treat age-associated diseases.

Our recent studies reveal important roles of the mitochondrial type-II arginase (Arg-II), a mitochondrial L-arginine-ureahydrolase that catalyzes the hydrolysis of L-arginine to L-ornithine and urea (Olivon et al., 2013) in the pathogenesis of many diseases, including cardiovascular diseases and metabolic diseases associated with cellular senescence and inflammation (Ming et al., 2012; Yepuri et al., 2012). Studies in vascular endothelial cells demonstrate that Arg-II promotes vascular endothelial senescence and dysfunction via oxidative stress, which results from uncoupling of the vascular protective endothelial nitric oxide synthase (eNOS), a situation in which eNOS generates superoxide anion instead of NO (Mori, 2007; Santhanam et al., 2008; Yepuri et al., 2012). Moreover, an increase in Arg-II expression induces vascular smooth muscle cell senescence and apoptosis (Xiong et al., 2013). This function of Arg-II is mediated through interaction with mTOR/S6K1 and p66^Shc^ in vascular cells, which contributes to atherosclerotic cardiovascular diseases and cardiovascular aging. Indeed, genetic knock-down or knock-out of Arg-II in cells or mice (Arg-II^−/−^) demonstrates beneficial effects on the prevention of atherosclerosis, vascular aging, and obesity- and age-associated glucose intolerance (Ming et al., 2012; Yepuri et al., 2012; Wu et al., 2015; Xiong et al., 2017). These beneficial effects of Arg-II deficiency on various cellular and organ functions prompted us to hypothesize that Arg-II deficiency could modulate lifespan in mice. Thus, the aim of our current study is to analyze whether genetic deficiency of Arg-II gene in mice has an impact on lifespan extension and whether this effect is associated with decreased surrogate aging makers, such as S6K1 activity, p66^Shc^ and p16^INK4a^ levels in various organs, including skin and heart.

## Materials and methods

### Materials

Anti-Arg-II antibody (SC-20151) was from Santa Cruz Biotechnology, anti-alpha smooth muscle actin antibody (ab7817) was from Abcam, anti-p66^Shc^ antibody (06-203) was from Merck Millipor, anti-S6 ribosomal protein (#2317) and anti-phospho S6-S240/244 (#5364) antibodies were purchased from Cell Signaling Technology.

### Mouse husbandry and life span study

Arginase II gene deficient mice (Arg-II^−/−^) were kindly provided by Dr. William (Shi et al., 2001) and backcrossed to C57BL/6J for more than eight generations. Genotyping was performed by polymerase chain reaction (PCR) as previously described (Ming et al., 2012). The WT and Arg-II^−/−^ offsprings (F2) from hetero/hetero (F1) cross were interbred to obtain WT and Arg-II^−/−^ mice (F3), respectively, for our experiments. The mice were maintained in conventional (not specific pathogen free, SPF) conditions: 23°C, 12-h light-dark cycle and fed a normal chow (energy content: 10.6% fat, 27.6% protein, and 57% carbohydrate, fiber 4.8%; Provimi Kliba NAFAG 3436) and had access to tap water *ad libitum*. Kaplan-Meier survival curves were established by calculating lifespan based on known birth and death dates, with differences between groups evaluated using the log-rank test (Selman et al., 2009). Experiments were performed on mice derived from 8 to 17 different litters (8–17 different cohorts). Early lifespan is defined as the mean lifespan of the 25% animals with the shortest lifespan within a genotype; Late lifespan is defined as the mean lifespan of the 25% animals with the longest lifespan within a genotype. Additional young (3–6 months) and old (18–24 months) mice were sacrificed after anesthesia with xylazine (10 mg/kg body weight, intraperitoneally) and ketamine (100 mg/kg body weight, intraperitoneally) for isolation of heart and dorsal skin tissues, which were subjected to immunoblotting for analysis of aging-related gene and protein expression. Animal work was approved by the Ethical Committee of Veterinary Office of Fribourg (Nr. 2011_13_FR), Switzerland and was performed in compliance with guidelines on animal experimentation at our institution.

### Immunoblotting

Tissue lysate preparation, SDS-PAGE, and immunoblotting, antibody incubation and signal detection were performed as described previously (Ming et al., 2012). Quantification of the signals was performed using NIH Image J1.49 software.

### Tissue immunofluorescence staining and confocal microscopy

After deparaffinization in xylene, hydration in ethanol, and antigen retrieval in Tris-EDTA buffer (10 mM Tris-base, 1 mM EDTA solution, 0.05% Tween 20, pH 9.0) in a pressure cooker, paraffin-embedded sections (7 μm) were blocked with 1% BSA in PBS for 1 h and then were incubated with the primary antibodies overnight at 4°C and then washed 3 × 5 min with PBS. The sections were subsequently incubated with fluorescence-labeled secondary antibodies at room temperature in the dark for 2 h, followed by counterstaining with 300 nmol/L DAPI (4'6-diamidino-2-phenyl-indole dihydrochloride, Invitrogen) for 2 min. The immunofluorescence signals were visualized under LEICA's DIM6000 Confocal microscope.

### Quantitative reverse transcription PCR (qRT-PCR) analyses

mRNA expression of p16^INK4a^, Arg-II and glyceraldehyde 3-phosphate dehydrogenase (GAPDH) was measured by two-step qRT-PCR as described previously (Ming et al., 2012). Total RNA was extracted from cells or tissues with Trizol Reagent (Molecular Research Center, Inc., Cincinnati, OH, USA) following manufacturer's protocol. cDNA was generated from 500 ng total RNA with a random primer. Real-time PCR reaction was performed with the iQ™ SYBR Green Supermix and iCycler system (Bio-Rad). mRNA expressions were normalized to the reference gene GAPDH. Following PCR primers of mouse origin (m) were used:

mp16^INK4a^-F: GAACTCTTTCGGTCGTACCC
mp16^INK4a^-R: GCAGA AGAGCTGCTACGTGAA
mArg-II-F: CCCCTTTCTCTCGGGGACAGAA
mArg-II-R: GGGCGTGACCGATAATGGT
mGAPDH-F: ACCCAGAAGACTGTGGATGG
mGAPDH-R: ACACATTGGGGGTAGGAACA

### Statistics

Data are given as mean ± SD. In all experiments, n represents the number of experiments or animals. Log-rank tests were used for the all time point survival curves. For early time points of lifespan study, Gehan-Breslow-Wilcoxon test were used. Statistical analysis was performed with Student's unpaired *t*-test or analysis of variance (ANOVA) with Bonferroni *post-hoc* test. Differences in mean values were considered significant at two-tailed *P* ≤ 0.05.

## Results and discussion

**Figure 1:**
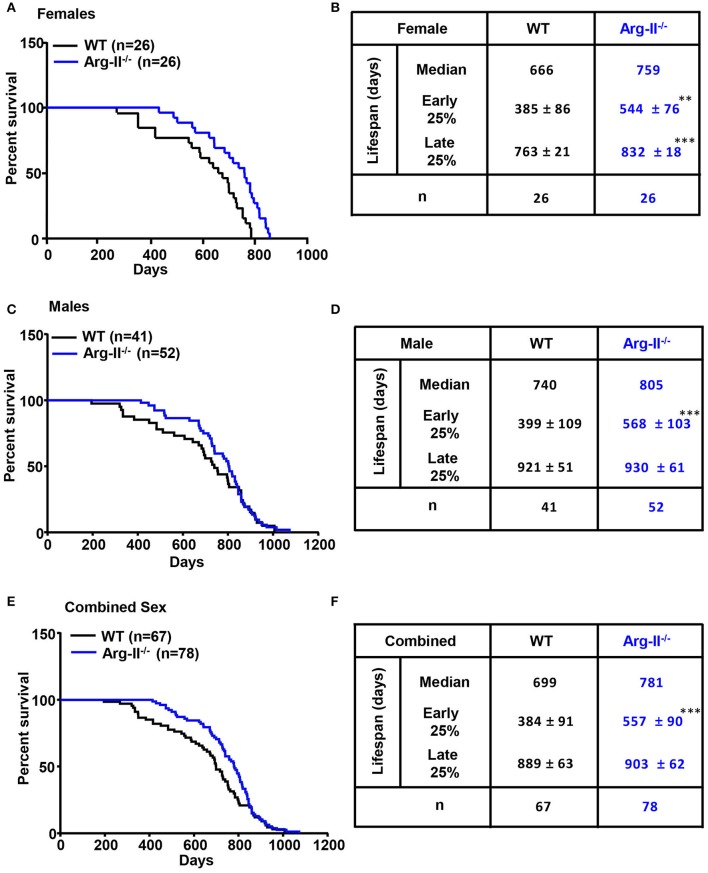
Effects of Arg-II ablation on mouse lifespan and Kaplan–Meier survival curve for female and male wild type (WT) and Arg-II^−/−^ mice. **(A)** Female Arg-II^−/−^ mice display significant lifespan extension (log-rank test, *p* < 0.01). **(B)** Comparative survival parameters of female WT and Arg-II^−/−^ mice. Early lifespan is defined as the mean lifespan of the 25% animals with the shortest lifespan within a genotype; Late lifespan is defined as the mean lifespan of the 25% animals with the longest lifespan within a genotype. **(C)** Male Arg-II^−/−^ mice show no significant increase in lifespan (log-rank test or Gehan-Breslow-Wilcoxon test, *P* > 0.05). **(D)** Comparative survival parameters of male WT and Arg-II^−/−^ mice. **(E)** Gehan-Breslow-Wilcoxon test for combined male and female WT and Arg-II^−/−^ mice show a significant (*p* < 0.01) lifespan extension in Arg-II^−/−^mice. **(F)** Comparative survival parameters of combined genders of WT and Arg-II^−/−^ mice. The values are mean ± SD. n indicates the number of animals of each experimental group. ^**^p < 0.01 and ^***^p < 0.001 vs. WT.

In this study, log-rank test is used to evaluate the difference in all time point lifespan of wild type (WT) and Arg-II^−/−^ mice on a C57BL/6 background (Ming et al., 2012; Yepuri et al., 2012). The results demonstrate a significant lifespan extension of Arg-II^−/−^ mice compared to WT controls in the female group (*P* < 0.001, Figure 1A). Median lifespan of Arg-II^−/−^ mice is increased by 93 days, from 666 to 759 days (Figure 1B). Early lifespan (mean lifespan of the 25% animals with the shortest lifespan within a genotype) and late lifespan (mean lifespan of the 25% animals with the longest lifespan within a genotype) are also increased in females (early lifespan: WT: 385 ± 86 days and Arg-II^−/−^: 544 ± 76 days, *p* < 0.01; late lifespan: WT: 763 ± 21 days and Arg-II^−/−^: 832 ± 18 days, *p* < 0.001; Figure 1B). In males, the effect of Arg-II ablation on overall lifespan extension does not reach statistical significance (Figure 1C), although median lifespan of male Arg-II^−/−^ mice is increased by 65 days from 740 to 805 days (Figure 1D), and early lifespan (not late lifespan) is significantly enhanced (*P* < 0.001, Figure 1D). For combined male and female WT and Arg-II^−/−^ mice, log-rank test does not show statistically significant effects on overall lifespan (Figure 1E). However, the early lifespan but not the late lifespan in Arg-II^−/−^ mice is significantly extended compared to WT controls (*P* < 0.001, Figure 1F). These results suggest a gender difference of Arg-II deficiency on lifespan regulation. Similar gender effect on lifespan was also reported in other genetically modified mouse models (Holzenberger et al., 2003; Selman et al., 2008, 2009). It is important to note that the mice were kept under conventional (not SPF) conditions, which resembles more closely our human living conditions. This explains a shorter lifespan of our mice compared to that reported in the literature.

**Figure 2:**
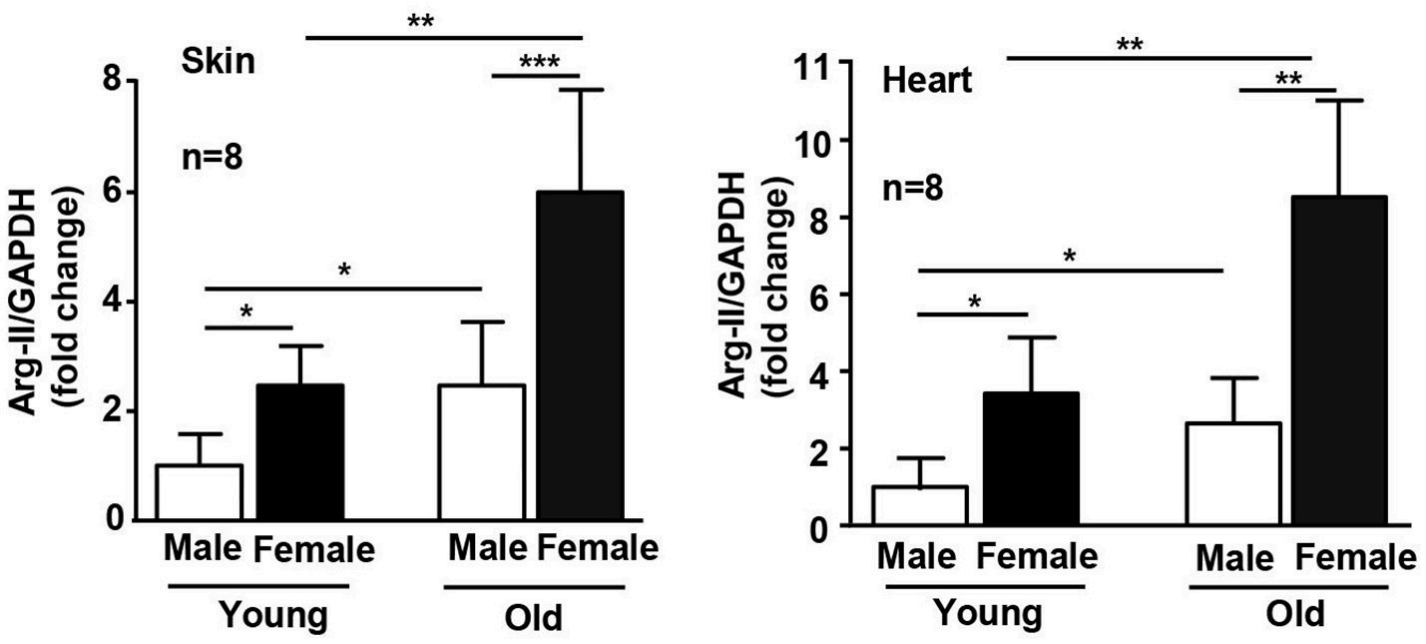
Age- and gender-associated difference in Arg-II expression. mRNA expression of Arg-II is analyzed by qRT-PCR in dorsal skin and heart of young and old WT mice. GAPDH serves as reference. The values shown are mean ± SD. n indicates the number of animals of each experimental group. ^*^p < 0.05, ^**^p < 0.01, and ^***^p < 0.001 between the indicated groups.

Interestingly, this gender difference is associated with differential Arg-II expression levels in WT females and males. The female mice have significantly higher Arg-II expression levels than the male mice in skin and heart at both young and old age (Figure 2). The impact of Arg-II on lifespan extension appears rather at an older age, since the early lifespan between the female WT and male WT mice does not differ significantly despite higher Arg-II expression in the young females, whereas the late lifespan of the female WT mice is shorter than that of the male WT mice. This may be due to a stronger elevation of Arg-II in the females than the males at an older age (Figures 1B,D). Accordingly, the effect of Arg-II deficiency on lifespan extension is more pronounced in the female mice. The underlying mechanism of gender-related differential Arg-II expression is not known and warrants further investigation. In a previous study, we reported that Arg-II deficiency in male mice on ApoE^−/−^ background leads to smaller aortic atherosclerotic plaques with more stable plaque features in comparison to the ApoE^−/−^ control mice (Ming et al., 2012). In the high fat diet-induced obesity model, the male Arg-II^−/−^ mice are protected from glucose intolerance (Ming et al., 2012). Taken together, these results and also the results from our current study demonstrate that Arg-II deficiency has significant influences on both males and females of the mouse models. Moreover, it becomes recognized that it is important to include male and female animals in biomedical research to avoid gender bias, which is clinically relevant.

**Figure 3:**
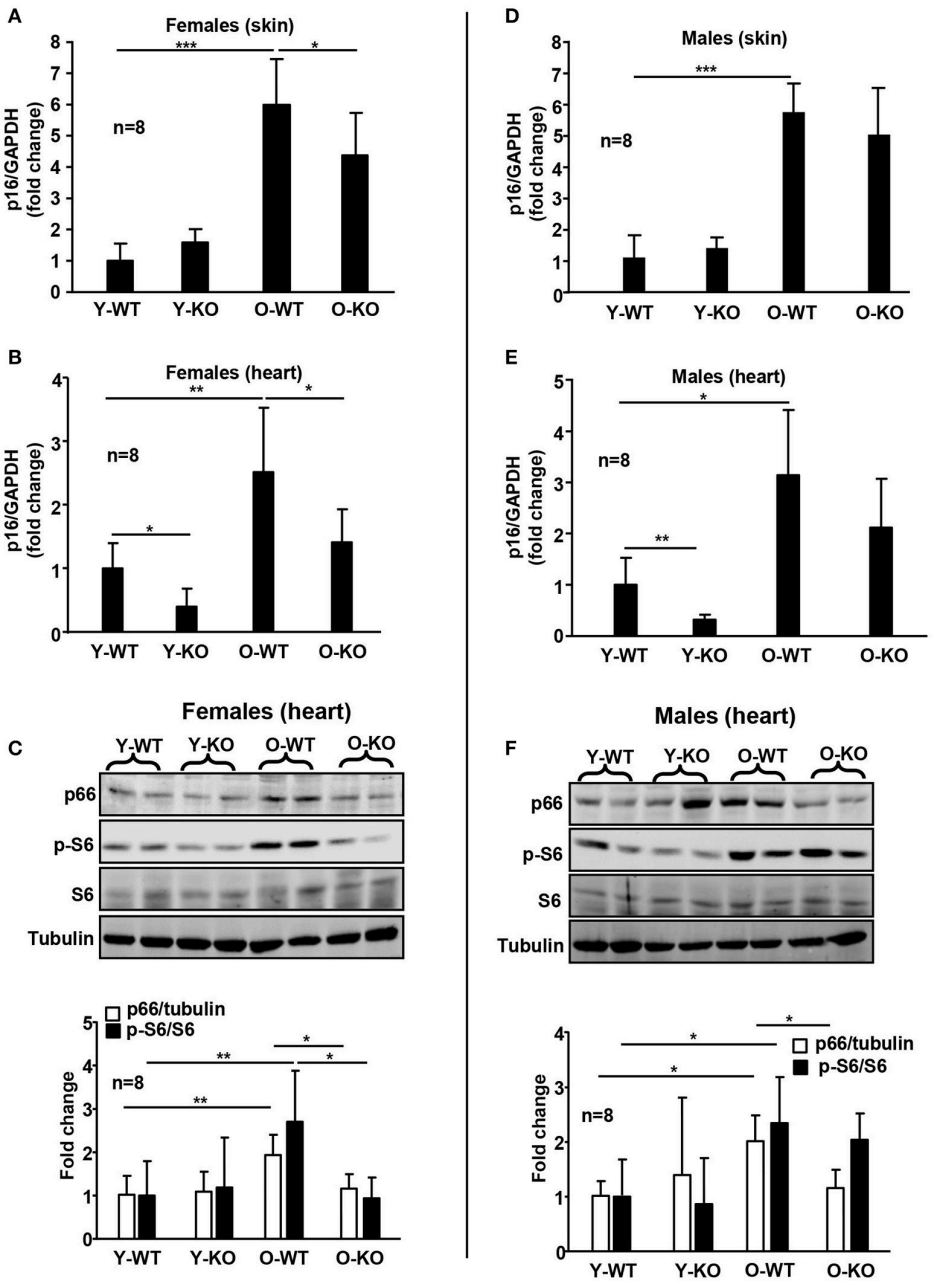
Deficiency of Arg-II reduces p16^INK4a^, p66^Shc^, and S6K1 in old mice. mRNA expression of p16^INK4a^ is analyzed by qRT-PCR in the dorsal skin **(A,D)** and heart **(B,E)** tissue of female and male mice. GAPDH serves as reference. Immunoblotting analyses of p66^Shc^, phosphorylated ribosomal S6 protein (S6-S240/244) and total S6 in heart from female **(C)** and male **(F)** WT and Arg-II^−/−^ mice. Tubulin serves as loading control. Y-WT, young wild type; Y-KO, young Arg-II^−/−^; O-WT, old wild type; O-KO, old Arg-II^−/−^. The values shown are mean ± SD. n indicates the number of animals of each experimental group. ^*^p < 0.05, ^**^p < 0.01, and ^***^p < 0.001 between the indicated groups.

The differential effect of Arg-II deficiency on lifespan extension in male and female mice is linked to the differential activation of signaling pathways that are involved in regulation of aging process or longevity. The mTOR (*M*echanistic *T*arget *O*f *R*apamycin) signaling pathway plays an essential role in regulation of lifespan and aging of organism (Johnson et al., 2013). Several studies showed that inhibition of mTOR signaling or of the down-stream target S6K1 extends lifespan in model organisms including mice (Selman et al., 2009; Lamming et al., 2012). Our recent study demonstrates that Arg-II promotes vascular cell senescence via mTORC1 and its downstream signaling S6K1, resulting in activation of mitochondrial p66^Shc^ (Xiong et al., 2013), a mitochondrial adaptor protein that generates intracellular ROS production and thereby shortens lifespan in mice (Migliaccio et al., 1999; Galimov, 2010). Moreover, cellular senescence and organism aging are characterized with enhanced p16^INK4a^ levels, and clearance of p16^INK4a^ cells extends lifespan in mice (Baker et al., 2016). In the present study, we observe a significant increase in p16^INK4a^ with aging in skin and heart of both female (Figures 3A,B) and male (Figures 3D,E) WT mice. Intriguingly, ablation of the Arg-II gene significantly reduces p16^INK4a^ levels in these tissues of old female mice (Figures 3A,B). In the male mice, this effect of Arg-II deficiency is weaker and does not reach statistical significance (Figures 3D,E). In line with this observation, age-associated increases in p66^Shc^ levels and S6K1 signaling in the heart of female mice are observed (Figure 3C), which is attenuated in the heart of female Arg-II^−/−^ mice (Figure 3C). In the male mice, Arg-II^−/−^ significantly reduces age-associated increase in p66^Shc^ levels in the heart, whereas S6K1 activation is not significantly reduced (Figure 3F). These results further support the gender biased effect of Arg-II deficiency on aging in female mice. Our results are in line with studies showing a more pronounced effect on lifespan extension in mice lacking S6K1 or with decreased mTORC1 activity in females (Selman et al., 2009; Lamming et al., 2012).

**Figure 4:**
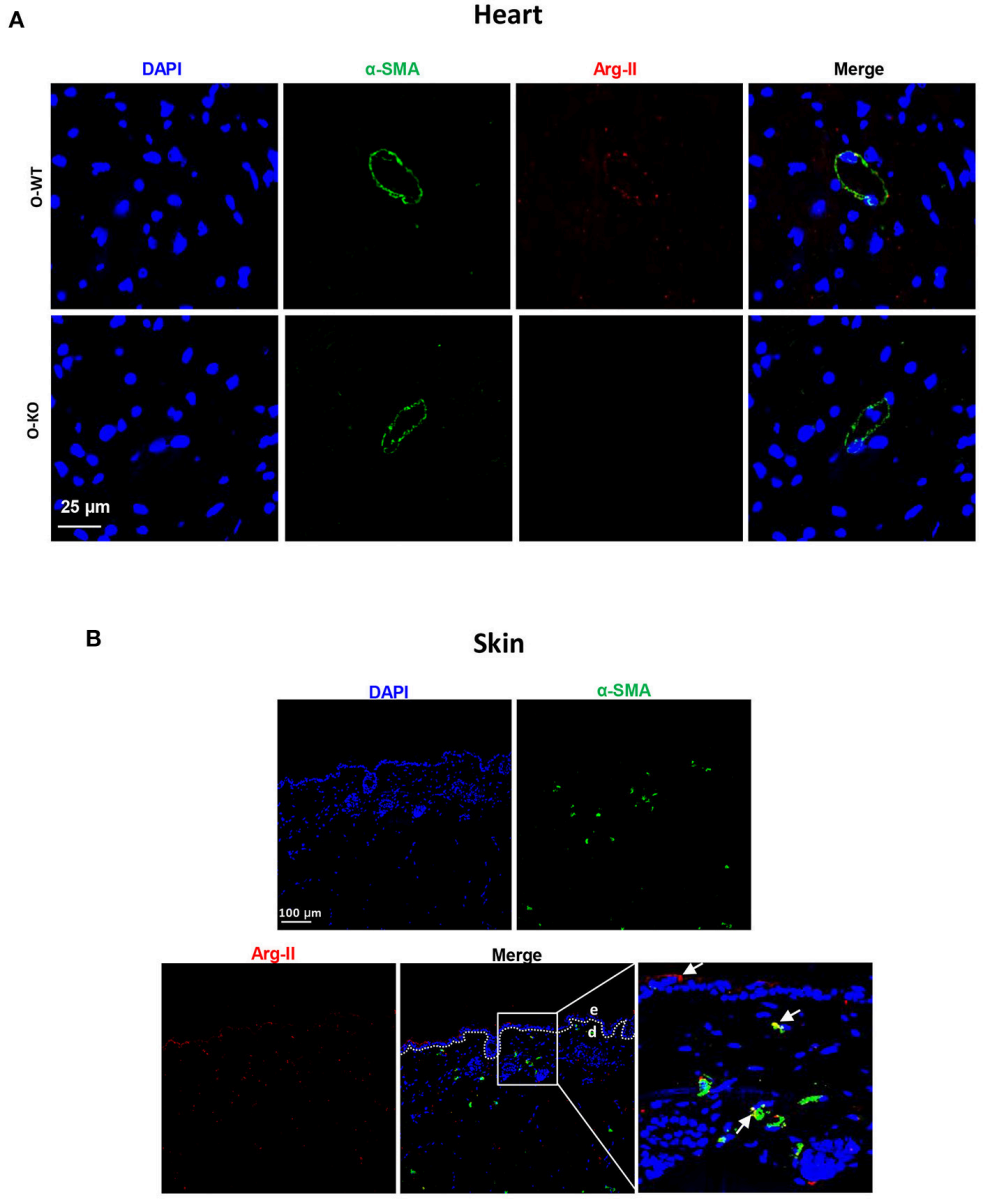
Immunostaining of Arg-II and α-smooth muscle actin (α-SMA) in an old WT mouse heart **(A)** and skin **(B)**. Arg-II (red) is detected in α-SMA positive cells as well as in α-SMA negative cells. Old Arg-II^−/−^ (O-KO) is used as negative control. The image in the right lower panel is the enlargement of the selected area. e, epidermis; d, dermis. Scale bar in **(A)**: 25 μm and in **(B)**: 100 μm.

Previous studies from other groups investigated functions of Arg-II in the heart of rodent animals. It was found that Arg-II is expressed in heart and located in cardiomyocyte mitochondria, where it regulates basal myocardial contractility (Steppan et al., 2006). We also found that Arg-II is not only expressed in small blood vessels, as evidenced by co-localization with α-smooth muscle actin (α-SMA) positive cells that form a circular morphology, but also expressed in other cells (Figure 4A) that are most likely cardiomyocytes as demonstrated by other groups (Steppan et al., 2006). In line with this study, our current work also found an age-associated increase in Arg-II expression in the heart (Figure 2), which was shown to contribute to age-associated myocardial functional decline through uncoupling of nitric oxide synthase (Khan et al., 2012). We also found an age-associated increase in Arg-II expression in skin (Figure 2). It has been reported that both Arg-I and Arg-II are present in skin. Arg-I is mainly expressed in bulbs of anagen follicles along with lower expression in the adnexal sebaceous glands and surface epithelium (Hochstedler et al., 2013), whereas Arg-II is mainly expressed in skin keratinocytes and fibroblasts (Debats et al., 2009). Our current work confirms the observation that Arg-II is expressed in keratinocytes and in fibroblasts (co-localized with α-SMA positive cells) but also in α-SMA negative cells, probably resident immune cells (Figure 4B). There are studies suggesting that enhanced arginase activity may impair skin wound healing through reduced nitric oxide bioavailability (Kavalukas et al., 2012). The function of Arg-I in skin is controversial. While some studies demonstrate a role of Arg-I in impairment of wound healing (Kampfer et al., 2003), others suggest that Arg-I is required for wound healing (Witte et al., 2002; Campbell et al., 2013). It seems that there might be isoenzyme specific functions in the skin. The function of Arg-II in skin is rarely investigated. One recent study demonstrated that Arg-II plays a role in skin keratinocyte senescence (Kim et al., 2017), which is in line with our finding of an increased Arg-II expression in skin of aged mice. The enhanced Arg-II in skin is associated with the surrogate marker of aging, i.e., p16^INK4a^. A role of Arg-II in promoting vascular cell senescence has been demonstrated in our previous studies (Xiong et al., 2013, 2014). These findings support a role of Arg-II in cellular senescence and organ and/or organism aging.

Regulation of lifespan of an organism is complex, involving multiple organs and cells. The age-associated increase in Arg-II expression was also observed previously in other tissues or organs, such as blood vessels and pancreas (Steppan et al., 2006; Khan et al., 2012; Yepuri et al., 2012; Xiong et al., 2013, 2017). These studies demonstrate that Arg-II plays a causative role in cellular senescence and in the organ functional decline. Previous studies including our own demonstrate beneficial effects of Arg-II deficiency in protection against development of cardiovascular diseases, insulin resistance, and diabetic complications (Ming et al., 2012; Yepuri et al., 2012; Huang et al., 2016; Xiong et al., 2017), which could contribute to the lifespan extension by Arg-II deficiency. It remains a great challenge to investigate which organ or cell and to which degree of the organ or cell contribute to the extension of lifespan. To address this question, specific organ or cell Arg-II knockout models are required, which is particularly important and interesting for future investigation.

There are important questions/limitations that shall be investigated/addressed in the future. First, lifespan of mice kept in SPF conditions shall be analyzed, although the experimental condition in conventional laboratories resembles more human living conditions. For example, mice deficient in p66^Shc^ reveal extended lifespan under SPF conditions (Migliaccio et al., 1999), but shortened lifespan under conventional conditions (Giorgio et al., 2016). Second, it is interesting to analyze the causes of death in our mice models, which requires long-term investigation. Finally, causal relationship of S6K1, p66^Shc^, and p16^INK4a^ in Arg-II deficiency-induced lifespan extension shall be investigated.

Nevertheless, our study provides evidence demonstrating that Arg-II plays a significant role in the acceleration of aging in mice. Genetic disruption of Arg-II in mouse extends lifespan mainly in females, which is related to inhibition of p16^INK4a^, p66^Shc^, and S6K1 signaling pathways. Thus, Arg-II may represent a promising target to slow down aging process and extend lifespan as well as to treat age-related diseases.

## Author contributions

YX and GY performed lifespan experiments; YX did the rest of the experiments; YX, ZY, and XM carried out the project design, analyzed the data, and prepared the figures. YX, JM, ZY, and XM discussed scientific concept, interpreted data, wrote the manuscript, and did manuscript editing. YX, ZY, and XM are the guarantors of this work and, as such, had full access to all the data in the study and take responsibility for the integrity of the data and the accuracy of the data analysis. All authors reviewed the manuscript.

### Conflict of interest statement

The authors declare that the research was conducted in the absence of any commercial or financial relationships that could be construed as a potential conflict of interest.
