# Supplementary material for: Arginase-II Deficiency Extends Lifespan in Mice
Source: Front Physiol. 2017 Sep 8;8:682. doi: 10.3389/fphys.2017.00682 (PMC5596098; doi:10.3389/fphys.2017.00682)
Supplement: Supplementary file 1 [file Image1.PDF]

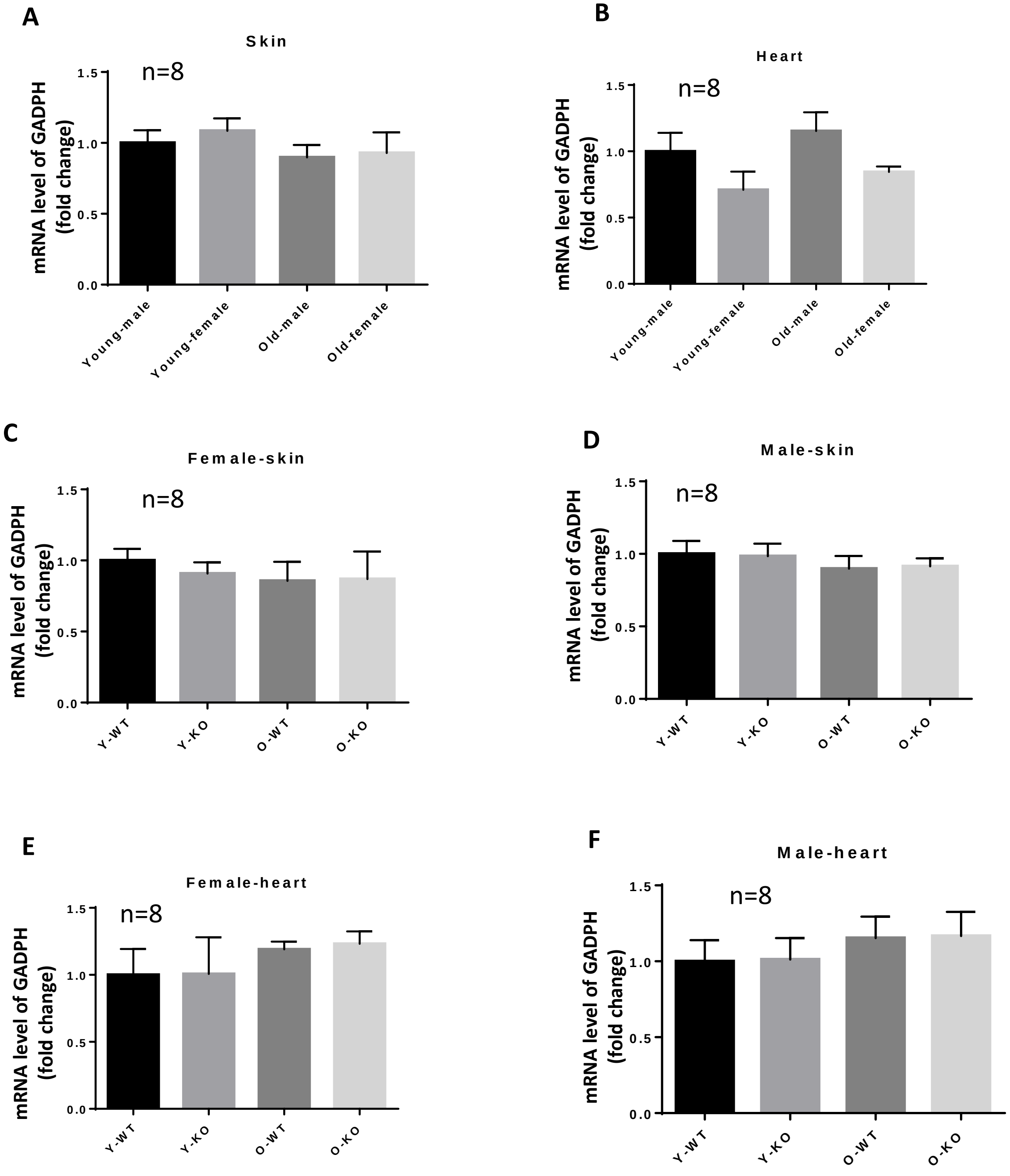

Supplement Figure 1 No significant age- and gender-associated difference in GAPDH mRNA levels. mRNA expression of GAPDH is analyzed by qRT-PCR in dorsal skin **(A)** and heart **(B)** of female/male young and old WT mice. mRNA expression of GAPDH is also analyzed by qRT-PCR in the female dorsal skin **(C)**, male dorsal skin **(D)**, female heart **(E)** and male heart **(F)** in the mice of Y-WT, Y-KO, O-WT and O-KO. Y-WT: young wild type; Y-KO: young Arg-II$^{-/-}$; O-WT: old wild type; O-KO: old Arg-II$^{-/-}$. The values shown are mean ± SD. n indicates the number of animals of each experimental group.
